# The efficacy and safety of Zuranolone for treatment of depression: A systematic review and meta-analysis

**DOI:** 10.1007/s00213-024-06611-y

**Published:** 2024-05-28

**Authors:** Aya M. Fayoud, Hisham Ahmed Orebi, Iman Abdelhady Elshnoudy, Mai Alaaeldin Temraz Elsebaie, Mariam Mahmoud Mohamed Elewidi, Hamdy Khaled Sabra

**Affiliations:** 1 Faculty of Pharmacy, Kafr El-Sheikh University, Kafr El-Sheikh, Egypt; 2 Medical Research Platform (MRP), Cairo, Egypt; 3 Faculty of Medicine, Tanta University, Tanta, Egypt; 4 Faculty of Medicine, Ain Shams University, Cairo, Egypt

**Keywords:** Zuranolone, Major depressive disorder, Postpartum depression, Synthetic neurosteroid

## Abstract

**Rationale:**

Zuranolone, a newly FDA-approved synthetic neurosteroid, shows promise in treating depression.

**Objectives:**

Our aim is to evaluate Zuranolone's efficacy and safety in treating depression.

**Methods:**

Five databases were searched until September 2023 for relevant randomized clinical trials evaluating the efficacy and safety of zuranolone. The potential risk of bias in the included trials was evaluated by the Cochrane Risk of Bias II guideline Data were extracted and pooled using Review Manager Software (RevMan 5.3).

**Results:**

An analysis of eight studies highlights Zuranolone's efficacy in treating depression compared to placebo across most of the outcomes. Notably, the 30mg and 50mg doses demonstrated significant improvements in reducing HAM-D scores by over 50% within a 15-day follow-up (RR) of 1.46 (95% CI [1.27, 1.68], *p* < 0.0001) and 1.14 (95% CI [1.01, 1.3], *p* = 0.04). Additionally, the HAM-D ≤ 7% score analysis revealed significant enhancements with the 30mg dose over both 15-day (RR = 1.82, 95% CI [1.44, 2.31], *p* < 0.0001) and 45-day (RR = 1.43, 95% CI [1.16, 1.77], *p* = 0.0008) durations. Adverse Events Drug Discontinuation demonstrated no overall significant difference (OR = 1.33, 95% CI: [0.79, 2.23], *p* = 0.282). Further, specific adverse events, such as headache, showed no significant overall difference between Zuranolone and placebo (OR = 1.11, 95% CI: [0.84, 1.47], *p* = 0.47), with dose-dependent analysis revealing less headache in the 30 mg group.

**Conclusion:**

Zuranolone demonstrates favorable tolerability and safety, particularly at 30mg and 50mg doses after 15 days, suggesting its potential and effective treatment for depression.

**Supplementary Information:**

The online version contains supplementary material available at 10.1007/s00213-024-06611-y.

## Introduction

Major Depressive Disorder (MDD) stands as a global mental health challenge, causing widespread disability (GBD 2017 Disease and Injury Incidence and Prevalence Collaborators 2018). Its manifestations involve alterations in affect, as well as cognitive, social, and occupational functions (Otte et al. 2016). The global prevalence of depression has surged, with over 19 million individuals in the United States having experiencing multiple depressive episodes, more than half of whom struggle with severe functional impairment (2019 NSDUH Detailed Tables n.d; Kessler et al. 2003). Unfortunately, approximately 788,000 individuals, burdened by depression, commit suicide (World Health Organization [Internet] 2017). Postpartum Depression (PPD), a form of major depressive disorder that emerges within four weeks after childbirth, leads to a decline in overall well-being and mental functioning (Bauman et al. 2020; Da Costa et al. 2006). The profound impact of PPD is evident through the loss of maternal-infant bonding and an increased susceptibility to suicide (Kerstis et al. 2016).

Treatment typically involves Selective Serotonin Reuptake Inhibitors (SSRIs), Serotonin-Norepinephrine Reuptake Inhibitors (SNRIs), and Tricyclic Antidepressants (TCAs) (Hockenberry et al. 2019). However, the effectiveness of these interventions is not guaranteed, with remission rate of over a 30% and at least 50% resistance to treatment with combined antidepressants(Kulkarni & Dhir 2009). After initial treatment, a significant number of patients struggle to maintain remission, contending with persistent symptoms that affect their quality of life and increase the risk of relapse (Trivedi 2009; Trivedi et al. 2006). Factors such as discontinuation of treatment and poor compliance, often due to delayed improvement or intolerance to side effects like weight gain and sexual dysfunction, contribute to this challenge (Bull et al. 2002; Geddes et al. 2003; Kulkarni & Dhir 2009).

The pathophysiology of depression is multifaceted, involving genetic, epigenetic, and environmental risk factors (Batterham et al. 2009). Disruption in the excitatory-inhibitory balance, regulated by glutamatergic and GABAergic signaling, is posited to play a role in depression development (Lener et al. 2017). This hypothesis gains support by observed alterations in GABA levels in the plasma, cerebrospinal fluid (CSF), and brain tissue of depressed patients, along with downstream changes in the expression of GABA-synthesizing enzymes and mRNA of GABA type A in individuals who died by suicide (Cutler et al. 2023; Gerner & Hare 1981; Luscher et al. 2011; Luykx et al. 2012; Merali et al. 2004; Sanacora et al. 2004).

Preclinical studies have identified allopregnanolone levels as a risk factor influencing GABAergic signaling (Osborne et al. 2017). Allopregnanolone, a neuroactive steroid and positive allosteric modulator of GABAA receptors, shows promise as an antidepressant, as evidenced by its normalization in CSF following SSRI treatment for depression (Paul & Purdy 1992; Uzunova et al. 1998).

Recently, Zuranolone, a synthetic neurosteroid and positive allosteric modulator of GABAA receptors, has gained approval from the U.S. Food and Drug Administration (FDA) for use in postpartum depression, based on two phase 3 randomized controlled trials (FDA NEWS RELEASE 2023; Heo 2023). Our goal is to systematically assess the efficacy and safety of Zuranolone for major depressive disorder and postpartum depression based on available clinical trials.

### Methodology

We followed the Preferred Reporting Items for Systematic Reviews and Meta-Analyses (PRISMA) statement guidelines (Page et al. 2021) and the Cochrane Handbook of Systematic Reviews and Meta-analysis (Higgins, et al. 2019). PRISMA checklist is illustrated in (See supplementary Tables S1 and S2, online resource).

### Data sources & search strategy

We searched PubMed, Web of Science, SCOPUS, clinicaltrials.gov and Cochrane Central through September 2023, using the following keywords ((Zuranolone OR SAGE$217) AND (Depress* OR dysphoria OR Melancholia OR dysthymi* OR "adjustment disorder*" OR "mood disorder*" OR "affective disorder*" OR "affective symptoms")).

### Eligibility criteria

We included clinical trials with the following PICO criteria: population (P): human patients with depression (e.g. MMD) or postpartum depression; intervention (I): Zuranolone; control (C): placebo. On the other hand, we excluded studies not fulfilling the previous criteria such as observational studies (cohort, case–control, cross-sectional, case series, and case reports), unpublished study protocols, letters to the editor, non-human studies, or those published in languages other than English.

### Selection process

Two authors independently carried out a two-step selection process, screening titles and abstracts of retrieved records. Subsequently, full texts of potentially eligible records were retrieved and assessed for inclusion in the meta-analysis. Any conflicts were resolved through discussion to reach a consensus.

### Data extraction

Four authors, using a pre-formed data extraction sheet, extracted the following data: study design characteristics (last authors' name, year of publication, NCT number, study design, country, inclusion criteria, exclusion criteria, intervention, control, outcomes, and Duration of treatment); Baseline sheet of the enrolled participants included study arms, number of participants in each arm, age in years, weight (kg), BMI (kg/m^2^), sex (%), ethnicity (%), race (%), Baseline antidepressant use No. (%) and baseline Hamilton Depression Rating Scale (HAMD) total score, mean (SD). All sheets were independently reviewed by the first author.

### Risk of bias

Two authors independently evaluated the potential risk of bias in the included trials following the Cochrane Risk of Bias II guideline (Sterne et al. 2019). We accordingly considered the following: randomization process, deviation from intended intervention, bias in the measurement of outcomes, selection of reported results, missing outcome data, and overall bias. Conflicts were resolved by reaching a consensus through discussion.

### Endpoints

#### Efficacy outcomes

HAMD-17 score improvement, Reduction of > 50% from baseline in HAM-D score, HAM-D ≤ 7% score, Clinical Global Impression Improvement (CGI-I) total score, Bech-6 total score, Montgomery-Åsberg Depression Rating Scale (MADRS) total score and Hamilton Rating Scale for Anxiety (HAM-A) total score.

#### Safety outcomes

Any treatment-emergent adverse event (TEAE), patients with a serious adverse event (any adverse event occurring while the patient was receiving the trial medication or placebo, that resulted in death, was immediately life-threatening, led to inpatient hospitalization or prolongation of hospitalization, caused persistent or clinically significant disability or incapacity, or resulted in a congenital abnormality or birth defect), patients with a severe adverse event (any event that was incapacitating or caused an inability to perform normal activities of daily living), adverse events drug discontinuation, and most common TEAEs.

### Statistical analysis

This meta-analysis was conducted by using two programs; Review Manager Software (RevMan version 5.3 n.d; Cochrane Collaboration, Copenhagen, Denmark, 2014) and Open Meta Analyst (OMA n.d) (Computer program) (Version 5.4. Copenhagen: The Nordic Cochrane Centre, the Cochrane Collaboration, 2014). We presented all data as either (1) mean difference (MD) in pooling continuous outcomes, or (2) odds/risk ratio (OR) in pooling dichotomous outcomes with 95% confidence intervals (CIs). We tested the heterogeneity between pooled studies using chi-square and I-square tests. When the heterogeneity between studies at chi-square of I^2^ > 50% P-value < 0.05, we used a random-effect model for analysis. We performed subgroup analysis to test whether the effect estimate of zuranolone differs significantly according to the dose and duration.

## Results

### Literature search results

**Fig. 1:**
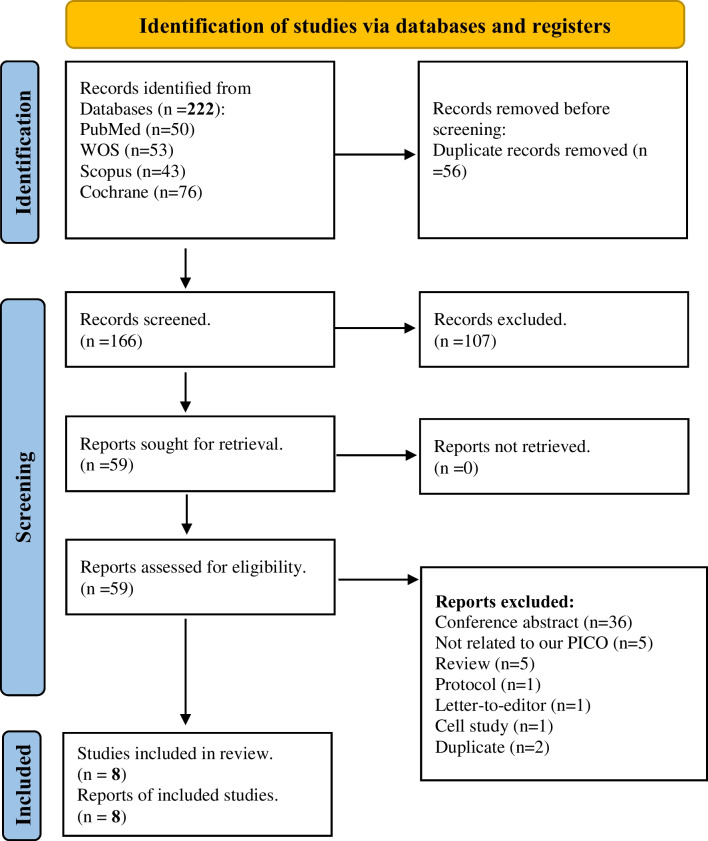
PRISMA flow diagram of the systematic review

Our systematic search identified 222 potential studies; Among these, 56 were excluded as duplicates. Following title and abstract screening, an additional 107 studies were excluded. Subsequently, full-text screening led to the exclusion of 51 studies. Finally, eight eligible studies were included for quantitative and qualitative synthesis in this systematic review. An extra 107 studies were excluded after title/abstract screening and then 51 studies were excluded after the full-text screening. In the end, we got eight eligible studies to be included in the quantitative and qualitative synthesis of this systematic review **(**Fig. 1; PRISMA).

### Characteristics of included studies

All the included studies were RCTs with a total number of 2,176 patients. The eight included RCTs were controlled with a placebo, with two of them incorporating two intervention arms featuring different doses of Zuranolone. Additionally, one study compared Zuranolone in combination with antidepressant therapy (ADT) to placebo with ADT. The number of patients in these included studies ranged from 89 to 537 patients with mean age varying between 27.4 and 49.1 years, and The Hamilton Depression Rating Scale ranged between 24.5 to 28.8. Zuranolone was administered orally once daily, with doses ranging from 20 to 50 mg over 2 weeks. Seven trials were conducted in the United States, and one study was conducted in Japan **(**Tables 1 and 2**).**

**Table 1:** Summary of included studies

Study ID	Protocol registration (NCT)	Study Design	Setting (country)	Total number of patients	Intervention	Comparison	Duration of treatment	Outcomes	Participants (inclusion criteria)	Exclusion criteria
Deligiannidis et al. 2021	NCT02978326	RCT	USA	150	Zuranolone (30-mg/day)	Placebo	14 days	Zuranolone was effective in improving symptoms of PPD indicated by HAMD-17 score, and generally well-tolerated	Female aged 18 to 45 years old, with PPD without psychosis (not more than 6 months postpartum)	Patients with known medical problems that prevent them from taking zuranolone
Gunduz-Bruce et al. 2019	NCT03000530	RCT	USA	89	SAGE 217 (30-mg/day)	Placebo	14 days	SAGE-217 was effective in reducing depressive symptoms, but the safety and tolerability is still questionable	Males and females aged 18 to 65 years old with MDD	Patients with history of suicide, resistant to antidepressant, or known medical problems that prevent them from taking the intervention
Clayton et al. 2023	NCT03672175	RCT	USA	482	Zuranolone (20-mg/day or 30-mg/day)	Placebo	14 days	Significant rapid improvements in depressive symptoms were observed with zuranolone 30 mg, and it was generally well-tolerated	Males and females aged 18 to 65 years old with MDD	Patients with history of suicide, resistant to antidepressant, or known medical problems that prevent them from taking the intervention
Deligiannidis et al. 2023	NCT02978326	RCT	USA	150	Zuranolone (30-mg/day)	Placebo	14 days	Zuranolone was effective in improving depressive, anxiety, and associated insomnia symptoms	Female aged 18 to 45 years old, with PPD without psychosis (not more than 6 months postpartum)	Patients with known medical problems that prevent them from taking zuranolone
Suthoff et al. 2022	NCT03000530	RCT	USA	89	SAGE 217 (30-mg/day)	Placebo	14 days	Zuranolone-treated patients reported rapid and significant improvements in HRQoL	Males and females aged 18 to 65 years old with MDD	Patients with history of suicide, resistant to antidepressant, or known medical problems that prevent them from taking the intervention
Kato et al. 2023a	JapicCTI-205276	RCT	Japan	249	Zuranolone (20-mg/day or 30-mg/day)	Placebo	14 days	Zuranolone was safe and demonstrated significant improvements in depressive symptoms	Males and females aged 18 to 75 years old with MDD	Patients with antidepressant resistance or known medical problems that prevent them from taking the intervention
Clayton et al. 2023	NCT04442490	RCT	USA	537	Zuranolone (50-mg/day)	Placebo	14 days	Zuranolone demonstrated significant improvements in depressive symptoms	Males and females aged 18 to 64 years old with MDD	Patients with history of suicide, resistant to antidepressant, or known medical problems that prevent them from taking the intervention
Parikh et al. 2024	NCT04476030	RCT	USA	430	Zuranolone (50-mg/day) + ADT	Placebo + ADT	14 days	Zuranolone demonstrated significant improvements in depressive symptoms	Males and females aged 18 to 64 years old with MDD	Patients with history of suicide, resistant to antidepressant, or known medical problems that prevent them from taking the intervention

*ADT* Antidepressant Therapy, *HAMD-17* 17-item Hamilton Rating Scale for Depression, *HRQoL* Health-related Quality of Life, *MMD* Major Depressive Disorder, *PPD* Postpartum Depression, *RCT* Randomized Controlled Trial, *USA* United States of America

**Table 2:** Baseline characteristics of the included studies

Study ID	Study Arm	No. of participants	Age, mean (SD), y	Sex No. (%)		Weight, mean (SD), kg	BMI, mean (SD), kg/m2	Ethnicity, No. (%)		Race, No. (%)				Baseline antidepressants use No. (%)	Baseline Hamilton Depression Rating Scale (HAMD) total score, mean (SD)
				Male	Female			Hispanic or Latino	Not Hispanic or Latino	African American	White	Asian	Other		
Deligiannidis et al. 2021	zuranolone 30 mg	76	29.3 (5.4)	0	100%	85.1(19)	31.1(6)	16 (21)	60 (79)	31(41)	44(58)		1(1)	16(21)	28.4(2)
	placebo	74	27.4(5.3)	0	100%	80.2(24)	30.3(8)	18(24)	56(76)	31(42)	40(54)		3(4)	13(18)	28.8(2)
Gunduz-Bruce et al. 2019	Zuranolone 30 mg	45	49.1(13.6)	20 (44)	25(56)	_	30.0(6.3)	_	_	36(80)	7(16)	1(2)	1(2)	12(27)	25.2(2.6)
	placebo	44	38.3(12.2)	14 (32)	30 (68)	_	29.9(5.2)	_	_	28(64)	16(36)	0	0	10(23)	25.7(2.4)
Clayton et al. 2023	zuranolone 30 mg	166	42.3 (11.8)	45 (27.1)	121 (72.9)	89.7 (22.4)	_	27 (16.3)	139(83.7)	64 (38.6)	94 (56.6)	2 (1.2)	6 (3.6)	47 (28.3)	25.9 (2.9)
	zuranolone 20 mg	159	41.9 (12.2)	47 (29.6)	112 (70.4)	87.3 (20.2)	_	31 (19.5)	128(80.5)	56 (35.2)	99 (62.3)	3 (1.9)	1 (0.6)	46 (28.9)	25.8 (2.8)
	placebo	157	41.4 (12.2)	51(32.5)	106 (67.5)	89.5 (22.9)	_	26 (16.6)	131(83.4)	54 (34.4)	96 (61.1)	3 (1.9)	4 (2.5)	49 (31.2)	25.8 (3.1)
Deligiannidis et al. 2023	zuranolone 30 mg	76	29.3 (5.4)	0	100%	85.1(19)	31.1(6)	16 (21)	60 (79)	31(41)	44(58)		1(1)	16(21)	28.4(2)
	placebo	74	27.4(5.3)	0	100%	80.2(24)	30.3(8)	18(24)	56(76)	31(42)	40(54)		3(4)	13(18)	28.8(2)
Suthoff et al. 2022	zuranolone 30 mg	45	49.1(13.6)	20 (44)	25(56)	_	30.0 (6.3)	_	_	36(80)	7(16)	1(2)	1(2)	12(27)	25.2(2.6)
	placebo	44	38.3(12.2)	14 (32)	30 (68)	_	29.9(5.2)	_	_	28(64)	16(36)	0	0	10(23)	25.7(2.4)
Kato et al. 2023a	placebo	82	40.8 (10.6)	35 (42.7)	47 (57.3)	63.4 (16.5)	23.6 (5.3)	0	82 (100)	0	0	82(100)	0	_	24.5 (2.1)
	zuranolone 20 mg	85	39.3 (12.6)	36 (42.4)	49 (57.6)	64.6 (13.8)	23.9 (4.4)	0	85 (100)	0	0	85(100)	0	_	24.8 (2.4)
	zuranolone 30 mg	82	38.8 (12.0)	35 (42.7)	47 (57.3)	61.0 (12.9)	22.7 (4.0)	0	82(100)	0	0	82(100)	0	_	24.6 (2.2)
Clayton et al. 2023	zuranolone 50 mg	268	39.4 (12.3)	82(30.6)	186 (69.4)	_	29.6 (6.3)	58 (21.6)	210 (78.4)	75(28.0)	169 (63.1)	13 (4.9)	11(4.1)	79 (29.5)	26.8 (2.6)
	placebo	269	40.1 (12.6)	103(38.3)	166 (61.7)	_	30.3 (6.2)	54 (20.1)	215 (79.9)	46 (17.1)	206 (76.6)	4 (1.5)	13(4.8)	81 (30.1)	26.9 (2.7)
Parikh et al. 2024	Zuranolone 50 mg + ADT	212	38.6 (12.72)	83 (39.2)	129 (60.8)	_	29.1 (6.26)	41 (19.3)	171 (80.7)	46 (21.7	153 (72.2)	6 (2.8)	3 (1.4)	115 (54.2)	26.8 (2.51)
	Placebo + ADT	218	37.7 (12.28)	78 (35.8)	140 (64.2)	_	29.9 (6.44)	52 (23.9)	166 (76.1)	31 (14.2	168 (77.1)	12 (5.5)	3 (1.4)	120 (55.0)	26.6 (2.58)

### Quality assessment

**Fig. 2:**
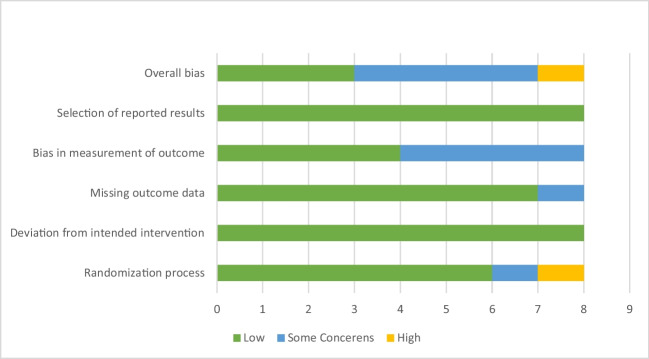
Risk of bias assessment of the included studies

According to the Cochrane Risk of Bias Assessment Tool for Randomized Clinical Trials II (ROB-II), the quality of the included studies ranges from low to some concerned risk of bias. One study showed a potentially high risk of bias in the randomization process domain. While four studies showed some concerned risk of bias regarding the measurement of the outcomes domain, and one study in the missing data domain (Fig. 2 and Table 3**).**

**Table 3:** Risk of bias assessment of the included studies

Domains	Risk of bias	Judgment of the authors
Deligiannidis et al. (2021)		
Randomization Process	Low	The randomization scheme was performed using an interactive response technology system vendor using SAS statistical software version 9.4 (SAS Institute). There were no baseline differences between intervention groups
Deviation from the intended intervention	Low	Participants and personnel were blinded
Missing outcome data	Low	Intention to treat analysis
Bias in the measurement of outcome	Low	All other site personnel except site–designated pharmacy staff were blinded to treatment assignments during the study
Selection of reported results	Low	The protocol was available, and data were analyzed in accordance with pre-specified plan
Overall bias	Low	The study is judged to be at low risk of bias for all domains
Gunduz-Bruce et al. (2019)		
Randomization Process	Low	Randomization was performed using interactive response technology created by 4G Clinical (Wellesley, MA). In a 1:1 ratio, patients were assigned to receive either SAGE-217 (30 mg) or placebo
Deviation from the intended intervention	Low	Participants and personnel were blinded, and analysis was appropriate
Missing outcome data	Low	Analyses were performed according to the intention-to-treat principle and included all patients who underwent randomization
Bias in the measurement of outcome	Some concerns	No information whether outcome assessors were aware of intervention or not and assessment can be influenced by knowledge of intervention
Selection of reported results	Low	The protocol was available, and data were analyzed in accordance with pre-specified plan
Overall bias	Some concerns	The study is judged to raise some concerns in one domain
Clayton et al. (2023)		
Randomization Process	Some concern	No information about the allocation concealment
Deviation from the intended intervention	Low	Participants and personnel were blinded
Missing outcome data	Some concerns	There is large number of follow up loss, but proportions of missing outcome data were balanced between intervention groups
Bias in the measurement of outcome	Some concerns	No information whether outcome assessors were aware of intervention or not and assessment can be influenced by knowledge of intervention
Selection of reported results	Low	The protocol was available, and data were analyzed in accordance with pre-specified plan
Overall bias	Some concerns	The study is judged to raise some concerns in three domains
Deligiannidis et al. (2023)		
Randomization Process	Low	The randomization scheme was performed using an interactive response technology system vendor using SAS statistical software version 9.4 (SAS Institute). There were no baseline differences between intervention groups
Deviation from the intended intervention	Low	Participants and personnel were blinded
Missing outcome data	Low	Intention to treat analysis
Bias in the measurement of outcome	Low	All other site personnel except site–designated pharmacy staff were blinded to treatment assignments during the study
Selection of reported results	Low	The protocol was available, and data were analyzed in accordance with pre-specified plan
Overall bias	Low	The study is judged to be at low risk of bias for all domains
Suthoff et al. 2022		
Randomization Process	Low	Randomization was performed using interactive response technology created by 4G Clinical (Wellesley, MA). In a 1:1 ratio, patients were assigned to receive either SAGE-217 (30 mg) or placebo
Deviation from the intended intervention	Low	Participants and personnel were blinded, and analysis was appropriate
Missing outcome data	Low	Analyses were performed according to the intention-to-treat principle and included all patients who underwent randomization
Bias in the measurement of outcome	Some concerns	No information whether outcome assessors were aware of intervention or not and assessment can be influenced by knowledge of intervention
Selection of reported results	Low	The protocol was available, and data were analyzed in accordance with pre-specified plan
Overall bias	Some concerns	The study is judged to raise some concerns in one domain
Kato et al. (2023a)		
Randomization Process	Low	Patients were randomized (1:1:1) at baseline (visit 1), with stratification based on the 17-item Hamilton Depression Rating Scale (HAMD-17) total score at baseline (< 25 vs ≥ 25) and sex, to receive zuranolone 20 mg, zuranolone 30 mg, or matching placebo once daily for 14 days (double-blind treatment period)
Deviation from the intended intervention	Low	Study was double blinded, and analysis was appropriate
Missing outcome data	Low	"Full analysis set (FAS) comprised all patients randomly assigned to the study drug and administered at least one dose of the study drug”; Analysis was intention to treat
Bias in the measurement of outcome	Some concerns	No information whether outcome assessors were aware of intervention, however; the assessment tools were reliable
Selection of reported results	Low	The protocol was available, and data were analyzed in accordance with pre-specified plan
Overall bias	Some concerns	The study is judged to raise some concerns in one domain
Clayton et al. (2023)		
Randomization Process	Low	Randomization, in a 1:1 ratio, was performed centrally via an interactive response technology system
Deviation from the intended intervention	Low	participants and personnel were blinded, and analysis was appropriate
Missing outcome data	Low	"543 were randomized, 537 were included in the safety set and 534 were included in the full analysis set", Nearly all randomized participants were involved in the analysis with missing outcome data is sufficiently small that their outcomes could have made no important difference to the estimated effect of intervention
Bias in the measurement of outcome	Low	Patients, clinicians, site personnel, the study sponsor, and the study team were blinded to treatment allocation. Blinding was maintained until database lock after all patients completed the study visit at day 42
Selection of reported results	Low	The protocol was available, and data were analyzed in accordance with pre-specified plan
Overall bias	Low	The study is judged to be at low risk of bias for all domains
Parikh et al. (2024)		
Randomization Process	High	Randomization was performed by stratification according to the co-initiated antidepressant and the administration of the antidepressants was open labelled
Deviation from the intended intervention	Low	Participants and personnel were blinded
Missing outcome data	Low	"Full analysis set (FAS) comprised all patients randomly assigned to the study drug and administered at least one dose of the study drug", Analysis was intention to treat
Bias in the measurement of outcome	Low	Outcome assessors were blinded
Selection of reported results	Low	The protocol was available, and data were analyzed in accordance with pre-specified plan
Overall bias	High	The study is judged to raise high risk of bias in one domain

### Outcomes

#### Efficacy outcomes

##### HAMD-17 score improvement

The overall effect estimates of 15-day follow-up duration showed a significant difference between Zuranolone and placebo groups favoring Zuranolone (SMD = -0.3, 95% CI: [-0.43, -0.17], *p* < 0.00001). The pooled results were heterogeneous (*p* = 0.03, *I*^*2*^ = 53%) which could not be solved. After introducing subgroup analysis based on Zuranolone dose, the effect estimates showed significant improvement in both Zuranolone 30-mg and Zuranolone 50-mg groups when compared to placebo group [(SMD = -0.44, 95% CI [-0.63, -0.24], *p* < 0.0001); *p* = 0.1, *I*^*2*^ = 49%] and [(SMD = -0.18, 95% CI [-0.31, -0.05], *p* = 0.008); *p* = 0.4, *I*^*2*^ = 0%], respectively. However, the effect estimate showed no significant difference in the Zuranolone group 20-mg when compared to the placebo group [SMD = -0.18, 95% CI [-0.45, 0.08], *p* = 0.18); *p* = 0.16, *I*^*2*^ = 49%] **(**Table 4). Also, see supplementary Fig. S1 (online resource).

**Table 4:** Mean difference OR standardized mean difference of the efficacy outcomes

Variables	Duration	Doses	Effect estimates			No. of participants		Heterogeneity	
			MD/SMD	95% CI	P	Zurnaonlone	Placebo	I2	P
HAMD-17	15 days	20-mg	-0.18	[-0.45, 0.08]	0.18	233	223	49	0.16
		30-mg	-0.44	[-0.63, -0.24]	< 0.0001	424	414	49	0.1
		50-mg	-0.18	[-0.31, -0.05]	0.008	437	466	0	0.4
		Total	-0.3	[-0.43, -0.17]	< 0.00001	1098	1103	53	0.03
	42–45 days	20-mg	-0.12	[-0.47, 0.022]	0.48	215	209	66	0.08
		30-mg	-0.26	[-0.45, -0.08]	0.004	401	396	38	0.17
		50-mg	-0.06	[-0.19, 0.08]	0.42	417	409	0	0.49
		Total	-0.16	[-0.28, -0.04]	0.008	1033	1014	42	0.09
Bech-6 score	NA	NA	-7.75	[-20.66, 5.15]	0.24	125	126	84	0.01
MADRS score	15 days	20-mg	-0.06	[-0.29, 0.17]	0.59	152	141	NA	NA
		30-mg	-0.31	[-0.46, -0.16]	< 0.0001	348	332	15	0.31
		50-mg	-0.2	[-0.38, -0.02]	0.03	247	250	NA	NA
		Total	-0.22	[-0.32, -0.12]	< 0.001	747	723	26	0.24
	42–45 days	20-mg	0.05	[-0.19, 0.28]	0.69	140	135	NA	NA
		30-mg	-0.24	[-0.4, -0.09]	0.002	331	322	39	0.18
		Total	-0.16	[-0.28, -0.03]	0.02	471	457	56	0.06
HAM-A score	15 days	20-mg	-1.03	[-2.99, 0.93]	0.3	232	223	28	0.24
		30-mg	-2.34	[-3.83, -0.85]	0.002	354	341	40	0.17
		50-mg	-1.29	[-2.41, -0.17]	0.02	247	250	NA	NA
		Total	-1.56	[-2.37, -0.74]	0.0002	833	814	25	0.24
	42–45 days	20-mg	-0.05	[-1.44, 1.34]	0.94	140	135	NA	NA
		30-mg	-2.42	[-5.63, 0.8]	0.14	258	253	76	0.01
		50-mg	-0.81	[-2.16, 0.54]	0.24	240	232	NA	NA
		Total	-1.33	[-2.83, 0.18]	0.08	638	620	66	0.02

CI: Confidence interval; HAM-A: Hamilton Rating Scale for Anxiety; HAMD-17: Hamilton Rating Scale for Depression-17; MADRS: Montgomery–Åsberg Depression Rating Scale; MD: Mean difference; SMD: Standardized mean difference

The overall effect estimates of 42 to 45-day follow-up duration showed a significant difference between Zuranolone and placebo groups favoring Zuranolone (SMD = -0.16, 95% CI: [-0.28, -0.04], *p* = 0.008). The pooled results were homogenous (*p* = 0.09, *I*^*2*^ = 42%). After introducing subgroup analysis based on Zuranolone dose, the effect estimates showed significant improvement in Zuranolone 30-mg when compared to the placebo group [SMD = -0.26, 95% CI [-0.45, -0.08], *p* = 0.004); *p* = 0.17, *I*^*2*^ = 38%]. However, the effect estimates showed no significant difference in both Zuranolone 20-mg and Zuranolone 50-mg groups when compared to the placebo group [SMD = -0.12, 95% CI [-0.47, 0.22], *p* = 0.48); *p* = 0.08, *I*^*2*^ = 66%] and [SMD = -0.06, 95% CI [-0.19, 0.08], *p* = 0.42); *p* = 0.49, *I*^*2*^ = 0%], respectively **(**Table 4). Also, see supplementary Fig. S2 (online resource).

##### Reduction of > 50% from baseline in HAM-D score

The overall effect estimates of 15-day follow-up duration showed a significant difference between Zuranolone and placebo groups favoring Zuranolone (RR = 1.25, 95% CI: [1.14, 1.36], *p* < 0.00001). The pooled results were homogenous (*p* = 0.05, *I*^*2*^ = 49%. After introducing subgroup analysis based on Zuranolone dose, the effect estimates showed significant improvement in both Zuranolone 30-mg and Zuranolone 50-mg groups when compared to the placebo group [(RR = 1.46, 95% CI [1.27, 1.68], *p* < 0.0001); *p* = 0.2, *I*^*2*^ = 33%] and [(RR = 1.14, 95% CI [1.01, 1.3], *p* = 0.04); *p* = 0.48, *I*^*2*^ = 0%], respectively. However, the effect estimate showed no significant difference between Zuranolone 20-mg when compared to the placebo groups [RR = 1.07, 95% CI [0.84, 1.37], *p* = 0.58); *p* = 0.34, *I*^*2*^ = 0%] **(**Table 5). Also, see supplementary Fig. S3 (online resource).

**Table 5:** Risk ratio of the efficacy outcomes

Variables	Duration	Doses	Effect estimates			No. of participants		Heterogeneity	
			RR	95% CI	P	Zurnaonlone	Placebo	I2	P
Reduction of > 50% from baseline in HAM-D score	15 days	20-mg	1.07	[0.84, 1.37]	0.58	233	223	0	0.34
		30-mg	1.46	[1.27, 1.68]	< 0.00001	426	413	33	0.2
		50-mg	1.14	[1.01, 1.3]	0.04	437	448	0	0.48
		Total	1.25	[1.14, 1.36]	< 0.00001	1096	1084	49	0.05
	42–45 days	20-mg	1.26	[0.76, 2.1]	0.36	215	209	77	0.04
		30-mg	1.24	[1.08, 1.43]	0.003	398	391	9	0.35
		50-mg	1.02	[0.82, 1.28]	0.84	417	409	71	0.06
		Total	1.16	[1.02, 1.33]	0.02	1030	1009	53	0.03
HAM-D score ≤ 7	15 days	20-mg	1.15	[0.78, 1.72]	0.48	233	223	51	0.15
		30-mg	1.82	[1.44, 2.31]	< 0.00001	426	413	6	0.37
		50-mg	1.19	[0.96, 1.48]	0.11	437	448	0	0.4
		Total	1.41	[1.21, 1.63]	< 0.00001	1096	1084	48	0.05
	42–45 days	20-mg	1.11	[0.79, 1.56]	0.54	215	209	0	0.75
		30-mg	1.43	[1.16, 1.77]	0.0008	398	391	53	0.07
		50-mg	1	[0.83, 1.21]	0.97	417	409	0	0.7
		Total	1.17	[1.03, 1.33]	0.02	1030	1009	47	0.06
CGI score of 1 or 2	15 days	20-mg	1.02	[0.8, 1.3]	0.16	151	141	NA	NA
		30-mg	1.34	[1.15, 1.57]	0.0002	273	259	38	0.2
		50-mg	1.59	[1.16, 2.16]	0.004	263	264	NA	NA
		Total	1.3	[1.15, 1.47]	< 0.0001	687	664	54	0.07
	42–45 days	20-mg	1.06	[0.83, 1.35]	0.66	140	135	NA	NA
		30-mg	1.22	[1.05, 1.42]	0.01	258	253	0	0.93
		Total	1.17	[1.03, 1.33]	0.02	398	388	0	0.77

*CI* Confidence interval, *CGI* Clinical Global Impression, *HAM-D* Hamilton Rating Scale for Depression, *RR* Risk ratio

The overall effect estimates of 42 to 45-day follow-up duration showed a significant difference between Zuranolone and placebo groups favoring Zuranolone (RR = 1.16, 95% CI: [1.02, 1.33], *p* = 0.02). The pooled results were heterogeneous (*p* = 0.03, *I*^*2*^ = 53%) which can’t be resolved. After introducing subgroup analysis based on Zuranolone dose, the effect estimates showed significant improvement in Zuranolone 30-mg when compared to the placebo group [RR = 1.24, 95% CI [1.08, 1.43], *p* = 0.003); *p* = 0.35, *I*^*2*^ = 9%]. However, the effect estimates showed no significant difference in both Zuranolone 20-mg and Zuranolone 50-mg groups when compared to the placebo group [RR = 1.26, 95% CI [0.76, 2.1], *p* = 0.36); *p* = 0.04, *I*^*2*^ = 77%] and [RR = 1.02, 95% CI [0.82, 1.28], *p* = 0.84); *p* = 0.06, *I*^*2*^ = 71%], respectively **(**Table 5). Also, see supplementary Fig. S4 (online resource).

##### HAM-D ≤ 7% score

The overall effect estimates of 15-day follow-up duration showed a significant difference between Zuranolone and placebo groups favoring Zuranolone (RR = 1.41, 95% CI: [1.21, 1.63], *p* < 0.00001). The pooled results were homogenous (*p* = 0.05, *I*^*2*^ = 48%. After introducing subgroup analysis based on Zuranolone dose, the effect estimates showed significant improvement in the Zuranolone 30-mg group when compared to the placebo group [RR = 1.82, 95% CI [1.44, 2.31], *p* < 0.0001); *p* = 0.37, *I*^*2*^ = 6%]. However, the effect estimates showed no significant difference in both Zuranolone 20-mg and Zuranolone 50-mg groups when compared to the placebo group [RR = 1.15, 95% CI [0.78, 1.72], *p* = 0.48); *p* = 0.115, *I*^*2*^ = 51%] and [RR = 1.19, 95% CI [0.96, 1.48], *p* = 0.11); *p* = 0.4, *I*^*2*^ = 0%], respectively **(**Table 5). Also, see supplementary Fig. S5 (online resource).

The overall effect estimates of 42 to 45-day follow-up duration showed a significant difference between Zuranolone and placebo groups favoring Zuranolone (RR = 1.17, 95% CI: [1.03, 1.33], *p* = 0.02). The pooled results were homogenous (*p* = 0.06, *I*^*2*^ = 47%). After introducing subgroup analysis based on Zuranolone dose, the effect estimates showed significant improvement in Zuranolone 30-mg when compared to the placebo group [RR = 1.43, 95% CI [1.16, 1.77], *p* = 0.0008); *p* = 0.07, *I*^*2*^ = 53%]. However, the effect estimates showed no significant difference in both Zuranolone 20-mg and Zuranolone 50-mg groups when compared to the placebo group [RR = 1.11, 95% CI [0.79, 1.56], *p* = 0.54); *p* = 0.75, *I*^*2*^ = 0%] and [RR = 1, 95% CI [0.83, 1.21], *p* = 0.97); *p* = 0.7, *I*^*2*^ = 0%], respectively **(**Table 5). Also, see supplementary Fig. S6 (online resource).

##### CGI-I total score

The overall effect estimates of 15-day follow-up duration showed a significant difference between Zuranolone and placebo groups favoring Zuranolone (RR = 1.3, 95% CI: [1.15, 1.47], *p* < 0.0001). The pooled results were homogenous (*p* = 0.07, *I*^*2*^ = 54%). After introducing subgroup analysis based on Zuranolone dose, the effect estimates showed significant improvement in both Zuranolone 30-mg and 50-mg Zuranolone groups when compared to the placebo group [RR = 1.34, 95% CI [1.15, 1.57], *p* = 0.0002); *p* = 0.2, *I*^*2*^ = 38%] and [RR = 1.59, 95% CI [1.16, 2.16], *p* = 0.004)], respectively. However, the effect estimates showed no significant difference in the Zuranolone 20-mg group when compared to the placebo group [RR = 1.02, 95% CI [0.8, 1.3], *p* = 0.87)] **(**Table 5). Also, see supplementary Fig. S7 (online resource).

The overall effect estimates of 42 to 45-day follow-up duration showed a significant difference between Zuranolone and placebo groups favoring Zuranolone (RR = 1.17, 95% CI: [1.03, 1.33], *p* = 0.02). The pooled results were homogenous (*p* = 0.77, *I*^*2*^ = 0%). After introducing subgroup analysis based on Zuranolone dose, the effect estimates showed significant improvement in Zuranolone 30-mg when compared to the placebo group [RR = 1.22, 95% CI [1.05, 1.42], *p* = 0.01); *p* = 0.93, *I*^*2*^ = 0%]. However, the effect estimates showed no significant difference in the Zuranolone 20-mg group when compared to the placebo group [RR = 1.06, 95% CI [0.83, 1.35], *p* = 0.66] **(**Table 5). Also, see supplementary Fig. S8 (online resource).

##### Bech-6 total score

The overall effect estimates showed no significant difference between Zuranolone and placebo groups (MD = -7.75, 95% CI: [-20.66, 5.15], *p* = 0.24). The pooled results were heterogenous (*p* = 0.01, *I*^*2*^ = 84%) which can’t be resolved **(**Table 4). Also, see supplementary Fig. S9 (online resource)

##### MADRS total score

The overall effect estimates of 15-day follow-up duration showed a significant difference between Zuranolone and placebo groups favoring Zuranolone (SMD = -0.22, 95% CI: [-0.32, -0.12], *p* < 0.0001). The pooled results were homogenous (*p* = 0.24, *I*^*2*^ = 26%). After introducing subgroup analysis based on Zuranolone dose, the effect estimates showed significant improvement in both Zuranolone 30-mg and Zuranolone 50-mg groups when compared to placebo group [(SMD = -0.31, 95% CI [-0.46, -0.16], *p* < 0.0001); *p* = 0.31, *I*^*2*^ = 15%] and [(SMD = -0.2, 95% CI [-0.38, -0.02], *p* = 0.03)], respectively. However, the effect estimate showed no significant difference in the Zuranolone 20-mg group when compared to the placebo group [SMD = -0.06, 95% CI [-0.29, 0.17], *p* = 0.59] **(**Table 4**). **Also, see supplementary Fig. S10 (online resource).

The overall effect estimates of 42 to 45-day follow-up duration showed a significant difference between Zuranolone and placebo groups favoring Zuranolone (SMD = -0.16, 95% CI: [-0.28, -0.03], *p* = 0.02). The pooled results were homogenous (*p* = 0.06, *I*^*2*^ = 56%). After introducing subgroup analysis based on Zuranolone dose, the effect estimates showed significant improvement in Zuranolone 30-mg when compared to the placebo group [SMD = -0.24, 95% CI [-0.4, -0.09], *p* = 0.002); *p* = 0.18, *I*^*2*^ = 39%]. However, the effect estimates showed no significant difference in the Zuranolone 20-mg group when compared to the placebo group [SMD = 0.05, 95% CI [-0.19, 0.28], *p* = 0.69] **(**Table 4). Also, see supplementary Fig. S11 (online resource).

##### HAM-A total score

The overall effect estimates of 15-day follow-up duration showed a significant difference between Zuranolone and placebo groups favoring Zuranolone (MD = -1.56, 95% CI: [-2.37, -0.74], *p* = 0.0002). The pooled results were homogenous (*p* = 0.24, *I*^*2*^ = 25%). After introducing subgroup analysis based on Zuranolone dose, the effect estimates showed significant improvement in both Zuranolone 30-mg and Zuranolone 50-mg groups when compared to placebo group [(MD = -2.34, 95% CI [-3.83, -0.85], *p* = 0.002); *p* = 0.17, *I*^*2*^ = 40%] and [(MD = -1.29, 95% CI [-2.41, -0.17], *p* = 0.02], respectively. However, the effect estimate showed no significant difference in the Zuranolone group 20-mg when compared to the placebo group [SMD = -1.03, 95% CI [-2.99, 0.93], *p* = 0.3); *p* = 0.24, *I*^*2*^ = 28%] **(**Table 4). Also, see supplementary Fig. S12 (online resource).

The overall effect estimates of 42 to 45-day follow-up duration showed no significant difference between Zuranolone and placebo groups (MD = -1.33, 95% CI: [-2.83, 0.18], *p* = 0.08). The pooled results were heterogeneous (*p* = 0.02, *I*^*2*^ = 66%). After introducing subgroup analysis based on Zuranolone dose, the effect estimates also showed no significant improvement in Zuranolone 20-mg, Zuranolone 30-mg, and Zuranolone 50-mg groups when compared to the placebo group [MD = -0.05, 95% CI [-1.44, 1.34], *p* = 0.94], [MD = -2.42, 95% CI [-5.63, 0.8], *p* = 0.14); *p* = 0.01, *I*^*2*^ = 76%] and [MD = -0.81, 95% CI [-2.16, 0.54], *p* = 0.24], respectively **(**Table 4). Also, see supplementary Fig. S13 (online resource).

#### Safety outcomes

##### TEAEs

For this outcome the higher the odd ratio (OR) the worse the outcome. Meaning more events occurred with the intervention. Looking at zuranolone, regardless of the dose, the intervention showed more TEAEs compared with the placebo (OR = 1.46, 95% CI: [1.15, 1.84], *p* = 0.002).). The pooled results were homogenous (*p* = 0.06, *I*^*2*^ = 46%). After introducing subgroup analysis based on Zuranolone dose, the higher the dose the more TEAEs reported, as for 50 mg, 30 mg vs 20 mg (OR = 1.71, 95% CI: [1.31, 2.22], *p* > 0.0001), (OR = 1.49, 95% CI: [0.93, 2.38], *p* = 0.09).), (OR = 1.19, 95% CI: [0.85, 1.66], *p* = 0.32), respectively. A dose of 50 mg showed a significant difference compared to the placebo. However, doses 30 mg and 20 mg showed non-significant differences **(**Table 6). Also, see supplementary Fig. S14 (online resource).

**Table 6:** Odds ratio of the safety outcomes

Variables	Doses	Effect estimates			No. of participants		Heterogeneity	
		OR	95% CI	*P*	Zurnaonlone	Placebo	I2	*P*
TEAEs	20-mg	1.19	[0.85, 1.66]	0.32	273	272	0	0.69
	30-mg	1.49	[0.93, 2.38]	0.09	275	462	64	0.02
	50-mg	1.71	[1.31, 2.22]	< 0.0001	480	487	0	0.42
	Total	1.46	[1.15, 1.84]	0.002	1228	1221	45	0.06
SAEs	20-mg	1.4	[0.274, 7.23]	0.682	273	272	0	0.836
	30-mg	1.46	[0.42, 5.313]	0.565	397	389	0	0.959
	50-mg	1.63	[0.31, 8.5]	0.562	480	487	0	0.374
	Total	1.49	[0.63, 3.54]	0.366	1150	1148	0	0.992
Severe AEs	30-mg	0.94	[0.21, 4.25]	0.936	123	117	0	0.983
	50-mg	2.05	[0.86, 4.87]	0.104	480	487	0	0.584
	Total	1.69	[0.8, 3.58]	0.17	603	604	0	0.783
AEs- related drug discontinuation	20-mg	0.66	[0.183, 2.38]	0.526	273	272	0	0.322
	30-mg	0.94	[0.36, 2.43]	0.906	475	462	0	0.874
	50-mg	2.01	[0.983, 4.09]	0.056	480	487	0	0.776
	Total	1.33	[0.79, 2.23]	0.282	1228	1221	0	0.735
Deaths	20-mg	1.93	[0.16, 23.1]	0.605	273	272	0	0.657
	30-mg	0.976	[0.14, 6.97]	0.98	397	389	0	1
	Total	1.27	[0.27, 5.92]	0.763	670	661	0	0.996
Headache	20-mg	1.66	[0.88, 3.13]	0.12	273	272	0	0.77
	30-mg	0.94	[0.56, 1.57]	0.81	379	389	0	0.77
	50-mg	1.04	[0.7, 1.56]	0.85	480	487	54	0.14
	Total	1.11	[0.84, 1.47]	0.47	1150	1148	0	0.61
Dizziness	20-mg	2.18	[1.04, 4.56]	0.04	273	272	0	0.91
	30-mg	1.76	[1, 3.11]	0.05	475	462	0	0.86
	50-mg	3.55	[0.98, 12.78]	0.05	480	487	82	0.02
	Total	2.33	[1.62, 3.35]	< 0.00001	1228	1221	9	0.36
Nausea	20-mg	1.13	[0.45, 2.85]	0.8	188	190	NA	NA
	30-mg	0.93	[0.3, 2.87]	0.9	315	307	44	0.17
	50-mg	0.32	[0.18, 0.57]	< 0.0001	212	218	NA	NA
	Total	0.71	[0.33, 1.52]	0.38	715	715	62	0.03
Somnolence	20-mg	1.65	[0.81, 3.38]	0.17	273	272	0	0.62
	30-mg	1.97	[1.24, 3.13]	0.004	475	462	0	0.6
	50-mg	3.58	[2.25, 5.7]	< 0.00001	480	487	66	0.09
	Total	2.48	[1.84, 3.33]	< 0.00001	1228	1221	21	0.26
Dry mouth	30-mg	2.16	[0.47, 9.92]	0.32	123	117	15	0.28
	50-mg	1.09	[0.56, 2.11]	0.8	212	218	NA	NA
	Total	1.23	[0.67, 2.23]	0.51	335	335	0	0.49
Sedation	20-mg	1.98	[0.75, 5.22]	0.166	273	272	0	0.803
	30-mg	1.83	[0.8, 4.19]	0.156	475	462	0	0.589
	50-mg	5.7	[0.6, 54.1]	0.13	480	487	75.56	0.043
	Total	2.29	[1.36, 3.84]	0.002	1228	1221	0.44	0.43
Decreased appetite	30-mg	0.48	[0.04, 5.46]	0.55	45	44	NA	NA
	50-mg	1.81	[0.7, 4.69]	0.22	212	218	NA	NA
	Total	1.5	[0.63, 3.56]	0.36	257	262	0	0.32
Insomnia	30-mg	0.48	[0.04, 5.46]	0.55	45	44	NA	NA
	50-mg	1.3	[0.67, 2.54]	0.44	212	218	NA	NA
	Total	1.2	[0.63, 2.29]	0.57	257	262	0	0.44
Diarrhea	20-mg	1.12	[0.46, 2.7]	0.8	188	190	NA	NA
	30-mg	1.26	[0.66, 2.39]	0.48	393	380	12	0.33
	50-mg	0.59	[0.34, 1.03]	0.07	480	487	0	0.88
	Total	0.86	[0.59, 1.26]	0.45	1061	1057	16	0.31
Upper respiratory tract infection	20-mg	1.48	[0.4, 5.45]	0.55	85	82	NA	NA
	30-mg	3.25	[1.27, 8.34]	0.01	238	228	0	0.49
	Total	2.54	[1.19, 5.38]	0.02	323	310	0	0.54
Fatigue	20-mg	0.6	[0.14, 2.55]	0.49	188	190	NA	NA
	30-mg	2.72	[1.05, 7.07]	0.04	270	263	0	0.96
	50-mg	1.75	[0.8, 3.79]	0.04	212	218	NA	NA
	Total	1.74	[1.01, 2.99]	0.04	670	671	0	0.4

*AEs* Adverse events, *CI* Confidence interval, *OR* Odds ratio, *SAEs* Serious adverse events, *TEAEs* Treatment emergent adverse events

##### Serious adverse events

The overall effect estimates showed a non-significant difference between Zuranolone and placebo groups (OR = 1.49, 95% CI: [0.62, 3.53], *p* = 0.366). After introducing subgroup analysis based on Zuranolone dose, the higher the dose the more serious adverse events reported, as for 50 mg, 30 mg vs 20 mg (OR = 1.62, 95% CI: [0.31, 8.5], *p* = 0.562), (OR = 1.46, 95% CI: [0.40, 5.313], *p* = 0.565), (OR = 1.40, 95% CI: [0.27, 7.23], *p* = 0.682), respectively. **(**Table 6). Also, see supplementary Fig. S15 (online resource).

##### Severe adverse events

Only two interventions (zuranolone 30, 50 mg) were included in this analysis. the overall effect estimates showed a non-significant difference between Zuranolone and placebo groups (OR = 1.69, 95% CI: [0.79, 3.58], *p* = 0.17). It is observed that the severe adverse events were more in dose 50 mg compared to dose 30mg (OR = 2.05, 95% CI: [0.86, 4.87], *p* = 0.104), (OR = 0.94, 95% CI: [0.20, 4.24], *p* = 0.936), respectively. **(**Table 6). Also, see supplementary Fig. S16 (online resource).

##### AEs-related drug discontinuation

The overall effect estimates showed a non-significant difference between Zuranolone and placebo groups (OR = 1.33, 95% CI: [0.79, 2.23], *p* = 0.282). After introducing subgroup analysis based on Zuranolone dose, the higher the dose the more adverse events drug discontinuation reported, as for 50 mg, 30 mg vs 20 mg (OR = 2.00, 95% CI: [0.98, 4.09], *p* = 0.056), (OR = 0.94, 95% CI: [0.36, 2.43], *p* = 0.906), (OR = 0.66, 95% CI: [0.18, 2.38], *p* = 0.526), respectively. **(**Table 6). Also, see supplementary Fig. S17 (online resource).

##### Most common TEAEs

Somnolence, dizziness, and headache were the most reported TEAEs in the zuranolone dose groups. However, the overall effect estimates showed a non-significant difference between Zuranolone and placebo groups (OR = 1.11, 95% CI: [0.84, 1.47], *p* = 0.47) for headache. In contrast, the zuranolone showed more dizziness compared with the placebo (OR = 2.33, 95% CI: [1.62, 3.35], p > 0.00001).) and Somnolence (OR = 2.48, 95% CI: [1.84, 3.33], p > 0.00001).). (Table 6). Also, see supplementary Fig. S18 (online resource).

Regardless of the dose, zuranolone showed more sedation (OR = 2.28, 95% CI: [1.36, 3.84], *p* = 0.002), fatigue (OR = 1.74, 95% CI: [1.01, 2.99], *p* = 0.04).), and upper respiratory tract infection (OR = 2.54, 95% CI: [1.19, 5.38], *p* = 0.02) compared to the placebo. In contrast, the overall effect estimate showed a non-significant difference between zuranolone and placebo in terms of insomnia, dry mouth, decreased appetite, nausea, diarrhea, and death [(OR = 1.20, 95% CI: [0.63, 2.29], *p* = 0.57), (OR = 1.23, 95% CI: [0.67, 2.23], *p* = 0.51), (OR = 1.50, 95% CI: [0.63, 3.56], *p* = 0.36), (OR = 0.71, 95% CI: [0.33, 1.52], *p* = 0.38), (OR = 0.86, 95% CI: [0.59, 1.26], *p* = 0.45), and (OR = 1.26, 95% CI: [0.27, 5.92], *p* = 0.763)], respectively. (Table 6). Also, see supplementary Fig. S18 (online resource).

## Discussion

Depression, a pervasive mental health challenge affecting millions globally, prompts a continuous search for more effective and safer treatment options (Reddy 2010) Zuranolone, a novel selective neuroactive steroid GABAA receptor-positive allosteric modulator, exhibits promise in preclinical and early clinical trials for depression treatment (Althaus et al. 2020) This meta-analysis consolidates the latest data from 8 randomized controlled trials (RCTs) exploring zuranolone's efficacy and safety in major depressive disorder (MDD) and postpartum depression (PPD).

Our study evaluates zuranolone's impact compared to placebo across varied follow-up durations, employing common outcome measures in depression research (Rabin et al. 2022). The primary goal is to determine zuranolone's overall efficacy while scrutinizing its safety profile based on reported adverse events (AEs). Noteworthy findings reveal significant benefits favoring zuranolone in terms of efficacy outcomes. Specifically, both the 30-mg and 50-mg zuranolone groups exhibit substantial improvement at the 15-day follow-up, reflected in a reduction of over 50% from baseline in the Hamilton Depression Rating Scale (HAM-D) score. A similar trend is observed at the 42 to 45-day follow-up.

## Efficacy outcomes

Unlike other common GABA_A_ positive allosteric modulators like benzodiazepines, zuranolone can modulate both synaptic and extrasynaptic GABA_A_ conductance due to binding to a non-benzodiazepine site on the receptor (Clayton et al. 2023; Stahl et al. 2023). Furthermore, it may restore the balance of GABA_A_ receptor function that is disrupted by the rapid decline of allopregnanolone after childbirth (Stahl et al. 2023).

The overall effect estimates demonstrated a statistically significant improvement in HAMD-17 following 15 days after zuranolone administration and with a 42 to 45-day follow-up duration. Subgroup analysis based on zuranolone dose revealed that both the zuranolone 30-mg and 50-mg groups showed significant improvement in HAMD-17 scores compared to the placebo group. This is consistent with the study conducted on women with PPD which reported that rates of concurrent remission of depressive and anxiety symptoms were higher with zuranolone versus placebo (Deligiannidis et al. 2023) also a study conducted in the United States among adults with MDD reported significant improvement at days 3, 8, and 12 (Clayton et al. 2023).

On the other hand, the effect estimate for the zuranolone 20-mg group did not show a significant difference compared to the placebo group. However, a study conducted on Japanese patients aged between ≥ 18 years and ≤ 75 years with a diagnosis of MDD to test the efficacy and safety of zuranolone (Kato et al. 2023a) reported improvement in HAMD-17 scores and insomnia symptom score showed nominally significant differences in the zuranolone 20 mg groups when compared with the placebo group at Day 15. This suggests that the efficacy of zuranolone may vary depending on the specific dose used, and the 20-mg dose may not be as effective in improving HAMD-17 scores.

Regarding the Bech-6 scale (a shortened version of the HAMD-17 scale), our pooled analysis showed a great numerical improvement in zuranolone compared to placebo, but this numerical improvement didn’t reach statistical significance. Our finding was consistent with Kato et al. and Gunduz-Bruce et al. (Gunduz-Bruce et al. 2019; Kato et al. 2023a). However, Gunduz-Bruce et al. showed statistical significance. Unfortunately, the Bech-6 scale was reported by two studies, however; it’s more sensitive than the HAMD-17 scale in detecting treatment effects. The small sample size and high heterogeneity in our pooled analysis may mask the statistical significance of the treatment effects (Dunlop et al. 2019).

Regarding the reduction of > 50% from baseline in HAM-D score, this meta-analysis demonstrates that zuranolone, particularly at the 30-mg dose, is associated with a higher likelihood of achieving a reduction of > 50% from baseline in HAM-D score at both the 15-day and 42 to 45-day follow-up durations. However, there was no significant difference between Zuranolone 20-mg when compared to the placebo groups at 15 days and 42 to 45-day follow-ups as reported by a study conducted among Japanese adults (Kato et al. 2023a) and a study conducted in the United States among adults with MDD (Clayton et al. 2023). The negative results with these doses suggest the antidepressant effects of zuranolone may be threshold-dependent, requiring a minimum effective concentration to be reached. The 20 mg dose appears to be below this threshold level to consistently achieve clinically important reductions in depression severity (Walkery et al. 2021).

Additionally, zuranolone at 50 mg dosage showed no significant difference regarding the reduction of > 50% from baseline in HAM-D score between the treatment and placebo groups at 42 to 45-day follow-up duration. The lack of a significant difference seen with 50 mg dose versus placebo at 6 weeks also aligns with potential non-linear pharmacokinetics. Higher concentrations do not necessarily confer additional benefits and may come with an increased risk of adverse effects (Deligiannidis et al. 2021; Lin et al. 2023a, b). The lack of effect of 50 mg zuranolone compared to 30 mg may be attributed to a lack of compliance to 50 mg due to increased incidence of side effects (Lin et al. 2023a, b).

The ability to demonstrate a sustained response to antidepressant drugs was limited with the duration of follow-up of included trials. Nevertheless, it's important to evaluate the benefit of maintenance of medications over time to determine how long patients with MDD should take antidepressants. Kato et al. (Kato et al. 2021)recommended at least 6 months of maintenance therapy of antidepressants to prevent relapse and treatment failure as they found that the antidepressant maintenance group has a lower relapse rate than the antidepressant discontinuation group in both 6 months and 1 year maintenance periods. Another meta-analysis conducted by Kishi et al. (Kishi et al. 2023)recommended that maintenance treatment with antidepressants should be continued for at least 18 months or at least one year as the sustained response to antidepressants was more in 15 and 18 months than in 1 year or less. In contrast, the included studies, investigating the efficacy of zuranolone, only gave it for two weeks and the follow-up duration was relatively short; about 45 days. Furthermore, the overall effect achieved at 45 days of follow-ups was less than the effect achieved at 15 days of follow-ups in terms of HAMD-17 score, reduction of > 50% in HAM-D score, HAM-D score ≤ 7, CGI-I score of 1 or 2, MADRS score, and HAM-A score. Further long-term clinical trials are needed to evaluate the efficacy of a longer period of zuranolone maintenance in preventing relapse.

### Safety outcomes

This analysis indicates a higher occurrence of treatment-emergent adverse events (TEAEs) in comparison to the placebo. Significantly, the relative risk of TEAEs rises with escalating zuranolone doses, particularly with the 50-mg dose posing the greatest risk compared to the placebo (Kato et al. 2023a) This dose-dependent safety profile aligns with zuranolone's pharmacokinetic properties, underscoring the need to consider dosage levels when assessing safety (Parikh et al. 2024). These findings are consistent with a study assessing the efficacy and safety of zuranolone among adults with major depressive disorder (MDD), reporting that 74.1% of patients who received zuranolone experienced at least one TEAE. While the 30-mg dose showed a nominally higher TEAE risk than the 20-mg dose, this difference did not reach statistical significance. This suggests that the safety risks associated with the 30-mg dose are comparable to those of the 20-mg dose, acknowledging the limitations of the included studies and sample sizes.

Concerning severe adverse events and adverse events related to drug discontinuation, the comparison between zuranolone and placebo groups yielded non-significant differences, indicating a similarity in the occurrence of severe adverse events between the two groups. This finding aligns with a study assessing the tolerability of zuranolone among U.S. patients (Hoffmann et al. 2020) This is supported by studies conducted by Sage Therapeutics and Biogen, where no notable difference was found in adverse events related to discontinuation of the drug between the zuranolone and placebo groups, and a separate study involving women with PPD (Deligiannidis et al. 2021; Sage Therapeutics and Biogen Announce Positive Pivotal, n.d.).

The most frequent adverse events include somnolence, dizziness, and fatigue, with no alarming safety concerns. This agrees with a study conducted among adults with MDD to assess the efficacy and safety of zuranolone (Parikh et al. 2024).

## Strengths and limitations

This study was conducted based on PRISMA guidelines and pooled the results according to the available clinical trials to assess the efficacy and safety of zuranolone in depression. However, our review suffers from some limitations, including the limited number of randomized trials, and subsequently small sample size. Additionally, some of the measured outcomes were heterogeneous due to variations between the included studies. Our meta-analysis didn’t separate patients with postpartum depression and major depressive disorder. Although we acknowledge the importance of meticulously separate studies of zuranolone in postpartum patients from those involving MDD to better elucidate any nuanced effects and implications within each population, Lin et al. reported that the subgroup analysis didn’t show significant differences in outcomes between these two categories(Lin et al. 2023a, b). Also, most of the included studies were conducted in the US, making the generalizability of these results questionable.

## Conclusion

In conclusion, these findings suggest zuranolone's potential as an effective treatment for depression, emphasizing the crucial need for cautious consideration regarding its safety. Notably, there is no significant difference in discontinuation rates between the zuranolone and placebo groups.

### Supplementary Information

Below is the link to the electronic supplementary material.

Supplementary file1 (DOCX 831 KB)

## Data Availability

All data are available upon reasonable request from the corresponding author.
