# Correction to: The efficacy and safety of Zuranolone for treatment of depression: A systematic review and metaanalysis

**DOI:** 10.1007/s00213-024-06642-5

**Published:** 2024-06-22

**Authors:** Aya M. Fayoud, Hisham Ahmed Orebi, Iman Abdelhady Elshnoudy, Mai Alaaeldin Temraz Elsebaie, Mariam Mahmoud Mohamed Elewidi, Hamdy Khaled Sabra

**Affiliations:** 1 Faculty of Pharmacy, Kafr El-Sheikh University, Kafr El-Sheikh, Egypt; 2 Medical Research Platform (MRP), Cairo, Egypt; 3 Faculty of Medicine, Tanta University, Tanta, Egypt; 4 Faculty of Medicine, Ain Shams University, Cairo, Egypt

**Correction to: Psychopharmacology**

10.1007/s00213-024-06611-y.

In the published paper, the Family name of the author Mariam Elewidi was incorrectly listed as “Elewid”. The correct spelling of the name is “Elewidi”.

The original article has been corrected.

